# The heart, a secondary organ in the control of blood circulation

**DOI:** 10.1113/EP091387

**Published:** 2023-12-21

**Authors:** Branko Furst, José González‐Alonso

**Affiliations:** ^1^ Department of Anesthesiology Albany Medical Center Albany New York USA; ^2^ Sport, Health and Exercise Sciences, Department of Life Sciences, College of Health, Medicine and Life Sciences Brunel University London Uxbridge UK

**Keywords:** cardiac function, circulation models, circulatory control, exercise, haemodynamics

## Abstract

Circulation of the blood is a fundamental physiological function traditionally ascribed to the pressure‐generating function of the heart. However, over the past century the ‘cardiocentric’ view has been challenged by August Krogh, Ernst Starling, Arthur Guyton and others, based on haemodynamic data obtained from isolated heart preparations and organ perfusion. Their research brought forth experimental evidence and phenomenological observations supporting the concept that cardiac output occurs primarily in response to the metabolic demands of the tissues. The basic tenets of Guyton's venous return model are presented and juxtaposed with their critiques. Developmental biology of the cardiovascular system shows that the blood circulates before the heart has achieved functional integrity and that its movement is intricately connected with the metabolic demands of the tissues. Long discovered, but as yet overlooked, negative interstitial pressure may play a role in assisting the flow returning to the heart. Based on these phenomena, an alternative circulation model has been proposed in which the heart functions like a hydraulic ram and maintains a dynamic equilibrium between the arterial (centrifugal) and venous (centripetal) forces which define the blood's circular movement. In this focused review we introduce some of the salient arguments in support of the proposed circulation model. Finally, we present evidence that exercising muscle blood flow is subject to local metabolic control which upholds optimal perfusion in the face of a substantive rise in muscle vascular conductance, thus lending further support to the permissive role of the heart in the overall control of blood circulation.

## Abstract (highlights)

- **What is the topic of this review?**
  The question whether the heart or the peripheral circulation is the main determinant of systemic blood flow is the subject of a longstanding debate: in this review, the main theories explaining blood circulation are discussed.
- **What advances does it highlight?**
  The limitations of the prevailing ‘cardiocentric’ view of blood circulation are identified and an alternative view is put forward whereby the periphery plays a key role in the control of the magnitude of blood flow to the heart and thus the coupling of peripheral and central circulations.

## 1 INTRODUCTION

It is generally assumed that William Harvey (1578–1657), the discoverer of circulation, ascribed the blood's movement to the pressure‐generating function of the heart (Harvey, 1952a). However, Harvey did not compare the heart to a pump since at the time of publication of his seminal work, *On the Motion of Heart and Blood* (1628), the concept of hydrostatic pressure did not exist (Siegel, 1967). While Harvey accepted that rhythmic respiratory movements of the thorax were responsible for forward flow of the blood through the lungs (Harvey, 1952b), he ascribed the blood's movement and distension of the heart chambers to the blood's vitality and ‘innate heat’. Twenty years after the publication of his monograph, Harvey gave the following response to the famed Parisian anatomist Jean Riolan who contested his discovery (Harvey, 1952c):

I do not believe that the heart is the fashioner of the blood; neither do I imagine that the blood has powers, properties, motion, or heat, as the gift of the heart … rather, the blood gives heat to the heart, as it does to all the other parts of the body … And in this way I view the native or innate heat as the common instrument of every function, the prime cause of the pulse among the rest.

Harvey also compared the blood flow through the arteries to the action of a syphon. For Harvey's contemporary Rene Descartes (1596−1650), the blood was no longer a living fluid but a mixture of materials and food particles which serve as fuel for the fire maintained by the heart. With Descartes and his followers, the concept of vital heat was reduced to a process of combustion, a mere physical–chemical event, and the heart to a mechanical pump which impels the blood, an inert fluid (Fuchs, 2001). In the 1850s, debates abounded in the medical literature amongst the proponents of the view that the heart's ‘force from behind’, that is, *vis á tergo*, is the sole ‘motor’ of blood's movement, and those who believed that a ‘capillary power’, or *vis á fronte*, acting centripetally from the periphery, was equally important. For example, in his address to the Physiological Section of the Medical Society in London, Thudichum maintained that, ‘…if there were no other force promoting the circulation of the heart, the heart of a whale would be required in the human chest to effect even a very slow and languid circulation’ (Thudichum, 1855). By the 19th century the concepts of *vis á tergo* and *vis á fronte* were still mentioned in the literature for historical reasons but were largely devoid of their original meaning. With increase in the knowledge base and the availability of sophisticated experimental apparatus, these forces became the subject of detailed physiological study. Fluid dynamics of the microvascular beds was first systematically investigated by Starling, who demonstrated experimentally that tissue fluid exchange is governed by the balance of hydrostatic and oncotic pressures across the capillary wall, which behaves like a semipermeable membrane, the former pushing the fluid out and the latter pulling the fluids in the direction of the lumen (the Starling principle) (Starling, 1896). Despite the fact that the heart as a pressure‐propulsion pump was recognized as the principal source of blood propulsion, numerous attempts were made to demonstrate its ability to generate suction which would contribute to its filling and enhance its output (reviewed by Brecher, 1958).

**FIGURE 1:**
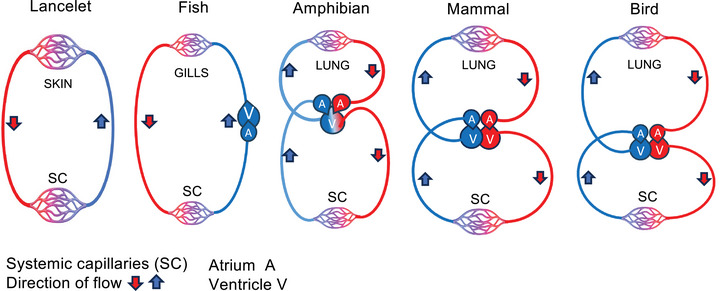
Evolutionary model of circulation: circulatory systems in early vertebrates, fish, amphibians and mammals. The lancelet, a primitive vertebrate, has no heart as a central organ of circulation and no gills, with the oxygen being absorbed through the skin. The blood moves autonomously within the vessels without endothelial lining. The fish have a single‐loop, predominantly venous circulation with a two‐chamber heart, a single atrium and a single ventricle, placed *in series* with the gill and systemic circulations. Transition from water to land called for development of a new organ, the lung, and for metamorphosis of the heart into a three‐chamber organ consisting of two atria and a single ventricle. In amphibians, arterial blood from the lung and venous blood from the body mix in the ventricle, which subserves low‐pressure pulmonary and systemic circulations placed *in parallel*. The circulatory system undergoes further development in warm‐blooded mammals with high metabolic rates and greater demands for oxygen. This is achieved by a complete separation of the pulmonary and systemic circulations. In addition to the existing ventricle serving the pulmonary circulation, a new chamber, the left ventricle, develops to serve the high‐pressure arterial circulation. The two circulations are placed *in series*. The cardio‐respiratory system in birds exemplifies a unique metabolic adaptation to extreme conditions at lower atmospheric pressures and temperatures and relative hypoxia (Scott, 2011). High metabolic rates reflected in physiological hyperthermia and hypertension have allowed the birds too overcome gravity and become creatures of air. (Adapted from Furst (2020a), used by permission of Springer‐Nature.)

However, the question whether the heart or the peripheral circulation is the main determinant of systemic blood flow continues to be the subject of ongoing debate. Proponents of the ‘cardiocentric’ view contend that the heart is the ‘motor’ for the circulating blood and the main determinant of cardiac output (CO) (Brengelmann, 2019; Levy, 1979; Reddi & Carpenter, 2005; Tyberg, 2002). The adherents of Guyton's venous return model, on the contrary, give priority to the peripheral circulation and ascribe to the heart a secondary or permissive role (Berger & Takala, 2018; Berger et al., 2016; Funk et al., 2013; Moller et al., 2017). Because the ultimate *source* for blood propulsion in *both* models is assumed to be the heart as a pressure‐propulsion pump, these opposing views differ only on the surface, not in essence, with little prospect for resolution. A systematic review of the circulation models has shown that neither the conventional ‘cardiocentric’ nor the alternative venous return circulation model can explain a host of circulatory phenomena (Furst, 2020a). For example, the debate over the source of blood propulsion in the valveless embryo heart (Männer et al., 2010) and in primitive vertebrates without a heart (Figure 1) continues to be unresolved; mechanical occlusion of the thoracic aorta results in a paradoxical increase, rather than decrease, in CO (Furst, 2020b) and the Fontan procedure, in which a single, weakened ventricle supposedly pumps the blood through the combined resistance of the systemic and pulmonary circulations, presents another, yet‐to‐be‐explained haemodynamic paradox (Furst, 2016, 2020c). The pressure‐propulsion paradigm is moreover at a loss to explain why a diverse group of conditions – ranging from clinically significant arterio‐venous fistulas to congenital heart abnormalities, that is, atrial and/or ventricular septal defects – should progress to Eisenmenger syndrome, if allowed to run their natural course (Furst, 2020d). The evolution of pharmacological treatment modalities for acute heart failure and cardiogenic shock over the past decades points to a radical shift in the understanding of the heart as a pressure‐propulsion pump. The widespread use of potent inotropes/vasoconstrictors such as adrenaline and isoproterenol in the 1960s and 1970s (Goldberg, 1968) has given way to short‐term use of inotropes with vasodilator properties (beta‐agonists, phosphodiesterase III inhibitors and calcium sensitizers) in a select group of patients (Bistola et al., 2019). Similarly, the use of intra‐aortic balloon pumps, based on the premise of afterload reduction and increase of CO, has fallen dramatically since several prospective studies failed to show an improvement in 30‐day mortality (up to 40%) in patients in cardiogenic shock (Baldetti et al., 2021).

An alternative circulation model has recently been proposed in which the peripheral circulation, responding chiefly to metabolic demands of the tissues and organs, plays a primary role in the control of CO (Alexander, 2017; Furst, 2020a). The heart, placed between the pulmonary and systemic circulations, integrates the metabolic, thermoregulatory and respiratory functions and provides a negative feedback control by rhythmically controlling the flow of blood and maintaining or altering perfusion pressure in the pulmonary and systemic circulations (Furst, 2015). Ontogenetically, the blood circulates before the functional maturity of the heart, indicating that flow is primary and pressure (pressure gradients) a secondary phenomenon. Given the heterogeneities in organ and tissue blood flow responses to physiological stressors including, but not limited to, exercise stress, hypoxia, hyperthermia and dehydration, the pressure gradients cannot be assumed to be the primary cause of the blood's propulsion. In this focused review we present the basic tenets of the pressure‐propulsion circulation model and highlight its inability to account for an increasing number of circulatory phenomena including exercise and pharmacologically induced hyperaemia.

## 2 PHYLOGENY OF THE CIRCULATION AND THE ROLE OF VASCULAR PRESSURE

Comparative biology of the cardiovascular system offers compelling examples against the pressure gradient generated by the heart being the primary driver of the circulation. It rather points to the primacy of flow as a response to the tissue's metabolic demands and the development of pressure in the arterial system as a later gain during vertebrate evolution (Furst, 2020e).

Invertebrates have an *open* circulation consisting of body cavities (coeloms) connected with vascular conduits without endothelial lining. This enables the circulating fluid, the haemolymph, to bathe the organs directly, thus serving a dual role of being a transport medium as well as a tissue fluid. In early aquatic vertebrates such as the lancelet (*Brachiostoma lanceolata*, Figure 1), the coelomic spaces have been transformed into primitive vessels lined with a simple epithelium. Lacking the basal lamina and thus being freely permeable to tissue fluid, the system is considered to be open (Rähr, 1981). The haemolymph contains cellular elements but lacks oxygen‐carrying pigments. There is no heart as the central organ of circulation and respiration occurs by absorption of oxygen through the skin. Pulsatile structures at the base of the branchial arches contract rhythmically and introduce pulsatile flow in the arteries. Fishes have a fully closed system of vessels complete with endothelial cells and basal lamina. The blood transports red blood cells (RBCs) containing haemoglobin. The gills and the systemic circulation are placed *in series* and the two‐chamber heart is positioned before the gills in the venous limb of the circuit. In a generic teleost, that is, trout, the gills are perfused at a higher pressure than the systemic circulation: 35 and 25 mmHg, respectively. Unlike in a mammalian heart, the ejection fraction in several teleost species is close to 100% (Sandblom et al., 2005). The discovery by Forouhar and co‐workers that the rate of flow in an embryonic zebrafish heart exceeds the velocity of the contractile wave sparked a yet‐to‐be‐resolved debate on the mechanism of blood propulsion in the embryonic vertebrate heart (Forouhar et al., 2006). Considering that the embryonic circulation still lacks the basement membrane and endothelial lining, it is conceivable that a pressure‐driven system would be ineffective on account of the seepage of haemolymph through the porous vascular wall. Several zebrafish *silent heart* mutants have been identified with a morphologically normal heart which, however, fails to contract. The embryos nevertheless show normal motility for up to 3 days (Mellish et al., 1994). Similarly, a variant of Mexican salamander (*Amblyostoma maxicanum*) has been described with a ‘cardiac lethal’ gene, whose larvae survive up to 2 weeks despite the non‐beating heart (Mellish et al., 1994).

In amphibians, the transition from a weightless watery environment to land called for the development of a primitive lung which supplements the vestigial oxygen absorption via the skin. A new atrium was added to the heart and the single ventricle now subserved the systemic and pulmonary circulations placed in parallel. Given that the venous blood from the systemic circuit and the oxygenated blood mix in the ventricle, it is difficult to explain how the pressure generated by the ventricle could equitably distribute an optimal amount of oxygenated blood to the systemic and pulmonary circulations without the presence of local flow control regulation (Joyce & Wang, 2021). In amphibians (as well as in fishes and mammals) this is achieved by a highly conserved process involving contraction of the vascular smooth muscle in the lung and dilatation at the periphery (Moudgil et al., 2005).

A complete separation of the pulmonary and systemic circulations is achieved in mammals where adjustment to gravity, air respiration, locomotion, inner reproduction and endothermy demands much higher tissue delivery of oxygen and nutrients than in ectotherms. The essential new feature of the mammalian circulation is a pressurized arterial system. The similarity of mean arterial pressures across the mammalian species suggests, however, that pressure does not affect blood propulsion but is indicative of environmental adaptation (Ivy & Scott, 2015; Penaloza, 2007).

The cardiovascular system in birds reaches yet another level of development. In comparison to mammals, birds have higher metabolic rates and are physiologically hyperthermic (40–42°C) and hypertensive. For example, reported direct arterial blood pressure values in awake and restrained red‐tailed hawks are in the range of 161–301 mmHg for systolic, and 142–245 mmHg for diastolic pressures, whereas in anaesthetized birds the values ranged from 124 to 251 mmHg for systolic and 78 to 198 mmHg diastolic pressures (Zehnder et al., 2009). Several bird species live in mountainous regions (above 4000 m) or fly at high altitudes (up to 11,000 m) and are subject to low atmospheric pressure and hypoxic conditions. In comparison to mammals with similar body mass, birds have a far more efficient lung O_2_ extraction – they have a one‐way, parabronchial air exchange system, versus a pooled alveolar system in mammals – larger hearts and greater stroke volumes. For example, studies on pigeons in a wind tunnel report rates of O_2_ consumption from basal of 18 ml kg^−1^ min^−1^ to 310 ml kg^−1^ min^−1^, with CO from 330 to 2244 ml kg^−1^ min^−1^ (Peters et al., 2005). In contrast, a trained 75 kg athlete consumes about 70–80 ml kg^−1^ min^−1^ of O_2_ at peak exertion and reaches CO of 27–35 litres min^−1^ or 360–466 ml kg^−1^ min^−1^ (e.g., Ekblom & Hermansen, 1968; Mortensen et al., 2005; Saltin & Åstrand, 1967). It is possible that high baseline arterial pressures protect the birds during sudden ascent to and descent from high altitudes, an environmental stress which is beyond endurance of most mammals.

In conclusion, the evolutionary development of the cardiovascular system shows that the flow is a primary and pressure a derived phenomenon related to the velocity of blood's movement, which, in turn, correlates with the metabolic tissue demands (Furst, 2015, 2020e). Where present and fully functional, the heart becomes an integral part of the cardiovascular system and the circulation cannot be sustained for prolonged periods without it. The developmental model demonstrates moreover that the height of arterial pressure is species specific and reflects environmental adaptation. As the evolutionary development of vascular systems passes through more rudimentary forms towards greater autonomy and specialization (Rosslenbroich, 2014), the key stages are repeated in a mammalian embryo, albeit in an accelerated form. The final transition occurs at birth when the predominantly venous, low‐pressure embryonic circulation supplied by the placenta changes to a high‐pressure, high‐O_2_‐content lung respiration.

## 3 VENOUS RETURN MODEL

**FIGURE 2:**
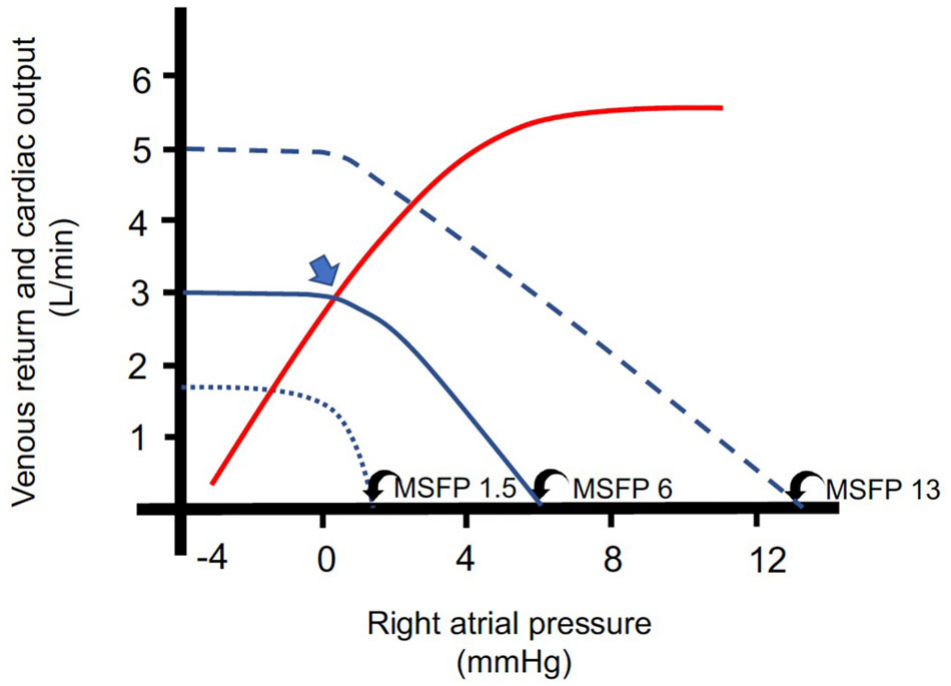
The effect of right atrial pressure (*P*_RA_) on venous return and cardiac output (CO). A family of venous return curves at normal, decreased and increased mean systemic filling pressure (MSFP) (continuous, dotted and dashed lines, respectively). Negative values of *P*_RA_ permit unhindered venous return and CO, as depicted by the horizontal portion of the curves (blue). With increasing values of *P*_RA_, venous flow diminishes and ceases when *P*_RA_ equals MSFP. The intersect of the normal venous return curve with the cardiac function (Frank–Starling) curve (red) represents the equilibrium point where venous return equals CO (blue arrow).

Between the 1950s and 1970s Arthur Guyton popularized the venous return model, which has gradually become widely accepted amongst basic scientists and clinicians and is featured in virtually every textbook of physiology (Guyton, 1968). It was conceived on the basis of seminal experiments by Frank and Starling on isolated heart preparations at the turn of the 19th century (Patterson & Starling, 1914). They observed that the myocardium is subject to autonomous regulation whereby an increase in ventricular filling is met with an increase in stroke volume and force of contraction. This fundamental property of the myocardium, the Frank–Starling relationship, can be represented on a graph relating CO as a function of the right atrial pressure (*P*_RA_) (red line, Figure 2).

Several phenomena convinced Guyton of the importance of the peripheral circulation in the overall control of CO, for example, that electrical pacing of the heart can more than double the heart rate, with little or no change in CO (Bada et al., 2012; Cowley & Guyton, 1971; Munch et al., 2014; Ross Jr et al., 1965; Stein et al., 1966). Cannulation of the superior and inferior vena cava in anaesthetized dogs and passing the blood through a perfusion pump before returning it to the heart failed to increase venous return even at progressively negative pump pressures (Guyton & Adkins, 1954). Similarly, a marked change in peripheral resistance – by creating an arteriovenous fistula (Huang et al., 1992) or an occlusion of the microvascular beds by injection of microspheres (Guyton, 1955) – exerted a relatively minor effect on CO, after a period of adjustment. In contrast, an increase in the circulating blood volume by incremental transfusion resulted in a sudden, proportional change in CO. On the basis of these phenomena, confirmed by a large amount of experimental data, Guyton and coworkers concluded that the peripheral circulation plays a dominant role in the control of CO (Guyton, 1968).

Guyton was aware of the fact that, while the Frank–Starling curves represent the behaviour of the heart at various values of *P*_RA_, they poorly reflect the properties of the systemic circulation. Given that around 70% of the blood volume is contained on the venous side (Boulpaep, 2003a), Guyton proposed that venous flow (*V*_F_) returning to the heart is the principal factor determining the heart's output. The concept of mean systemic filling pressure (MSFP), defined as pressure in the entire circulatory system after arrest of the heart during a no‐flow state, was adopted as a surrogate of the systemic vascular volume. The magnitude of the MSFP critically depends on blood volume and venous compliance, that is, the sympathetic tone (Magder, 2016). The theory was tested on open‐chest anaesthetized dogs in which the blood returning to the right atrium was passed through an adjustable resistance to a bypass pump and returned to the pulmonary artery (Guyton, 1963). A reverse relationship between *P*_RA_ and *V*_F_ was demonstrated and represented graphically as venous return curves (Guyton et al., 1957) (blue lines, Figure 2). In the mathematical formulation of the model, Guyton took the difference between MSFP and *P*_RA_ to represent the driving force for venous return:

(1) $$\mathrm{MSFP}-P_{\mathrm{RA}}=V_{F}\times R_{v}$$

where *R*_V_ is resistance, and (MSFP − *P*_RA_) the gradient for venous return.

When *V*_F_ equals CO, it follows from Ohm's law for fluids that:

(2) $$\mathrm{CO}=V_{F}=\left(\mathrm{MSFP}-P_{\mathrm{RA}}\right)/R_{v}$$

The gradient for venous return was therefore identified as the main determinant in the control of CO; viewed from the heart, the *P*_RA_ controls the degree of filling of the heart and regulates its output according to the above‐mentioned Frank–Starling relationship; seen from the perspective of the peripheral circulation, a higher *P*_RA_ exerts a back pressure and *impedes* venous return.

Since during a steady state venous return must equal CO, Guyton graphically superimposed venous return curves on cardiac function curves in a composite diagram to show the reciprocity between cardiac and venous functions (Figure 2). Such a representation of the cardiac and circulatory functions demonstrates the priority of venous return and confirms the permissive role of the heart, which ‘ejects all of the blood it receives’, as defined in the original formulation of Starling's law (Patterson & Starling, 1914) and in agreement with ideas proposed by Krogh around the same time (Joyce & Wang, 2021; Krogh, 1912a, 1912b). Despite the fact that Guyton's model was originally formulated for steady states, it has since been used to interpret dynamic changes in venous return (*V*_F_) and CO in the clinical treatment of heart failure in patients with circulatory assist devices (Kakino et al., 2017). Because the blood flow, that is, CO in extracorporeal membrane oxygenation (ECMO), is directly proportional to the pressure gradient for venous return (cf. Equation 2), this model can be used for the independent control of *P*_RA_ and pulmonary flows, allowing for a rational application of volume expansion and vasoconstrictors (Moller et al., 2019). The physiology of venous return and its relationship with the right heart function moreover provides a valuable framework for the understanding of compensatory mechanisms and therapeutic interventions in critical illness including septic, cardiogenic and hypovolaemic shock (Funk et al., 2013).

## 4 CRITIQUE OF VENOUS RETURN MODEL

Over the years, several critiques have been levelled against the venous return model. Levy repeated the above‐mentioned experiment by Guyton (without the resistive element between the right atrium and the bypass pump) and found an *inverse* relationship between *P*_RA_ and pump flow, that is, as the pump flow increased, *P*_RA_ decreased (Levy, 1979). Levy maintained that under the conditions of the experiment, venous return is clearly the dependent variable, and pointed to the liberal use of dependent (venous return and CO) and independent (*P*_RA_) variables in Guyton's analysis (Figure 2). Levy maintained that Guyton's idea that components of a model represent the actual counterparts of the cardiovascular system gives the impression that the *P*_RA_, rather than the bypass pump, controls CO. Such circular reasoning, according to Levy, amounts to a reversal of cause and effect (Levy, 1979). Unlike the arterial side, where a fall of arterial pressure, integrated by the baroreceptors, provides a clear negative feedback error signal because it represents a mismatch between the blood supplied by the heart and its demand by the tissues, the concept of ‘venous return’ is imprecise, lacks a clear error signal and should be abandoned (Reddi & Carpenter, 2005). In an attempt to ‘rescue’ the venous return model, Brengelmann contends that the (MSFP − *P*_RA_)/*R*_v_ concept (Equation 1) gives the erroneous impression that MSFP rather than the heart is the source of blood propulsion and maintains that Guyton arbitrarily chose to express flow‐dependent distending pressures relative to *P*_RA_ instead of the arterial pressure (Brengelmann, 2019). Finally, Tyberg suggests that both Guyton's and Levy's interpretations are model‐based and internally consistent, and it is ‘difficult or perhaps impossible to ‘prove’ one at the expense of the other’ (Tyberg, 2002).

## 5 ROLE OF THE HEART

Given that the heart is an integral part of the cardiovascular system, its mechano‐energetic properties have been difficult to assess. Several indices of myocardial performance have been proposed, such as ejection fraction, maximal velocity of shortening, peak isovolumetric pressure (d*P*/d*t*_max_) and stroke work index, to name but a few; however, none of these directly relate to the amount of blood the heart ejects against a given (aortic) pressure. To quantify the pump function of the ventricle, Elzinga and Westerhof tested an isolated feline heart preparation against a hydraulic model which closely mimics the behaviour of the arterial tree (Westerhof et al., 2009). The heart was stimulated at a fixed rate and maintained at a constant diastolic filling and contractility, while ejecting against variable aortic loads obtained by altering the resistance and compliance of the model arterial tree. The results showed that when ejecting against increased loads, that is, aortic pressures, the isolated heart generated smaller stroke volumes, until the flow ceased at maximal pressure (the isovolumic state), whereas with a gradual decrease in the aortic pressures, flows increased. The external power generated by the heart, namely, the product of pressure and flow, is therefore negligible at both extremes and is maximal at some intermediate value of flow (CO). This is the value at which the heart ejects during normal physiological arterial loads and is known as the ‘working point’. Of note, at each chosen flow and pressure, the myocardium ejected with *maximal* power and *efficiency* (expressed as the ratio of external work and oxygen consumption) (Elzinga & Westerhof, 1991). The fact that at a steady state the heart works at a maximal power has also been demonstrated on anaesthetized cats (Toorop et al., 1988) and in humans (Asanoi et al., 1989) but the underlying physiological principle by which the heart ‘chooses’ to work with optimal external power remains an open question. As mentioned, the arterial pressure in most mammals is approximately the same and the CO is adjusted to match the metabolic demands of the tissues. For a given arterial pressure there are numerous flows (COs) by which the heart could work to achieve its ‘working point’, but there is only one setting at which the external power is optimal for a particular heart. This setting is thought to correspond to optimal delivery of oxygen to the tissues in a particular animal or human at a given physiological state (Van den Horn et al., 1985).

In terms of energetics, the basal (non‐beating) metabolic rate of the myocardium is significantly higher compared to that of the skeletal muscle. For example, the resting metabolic rate in a rat skeletal muscle is 2–4 mW g^−1^, while the metabolic rate of the arrested rat heart at the same temperature is 20 mW g^−1^. A beating human heart produces an approximately 10 times greater amount of metabolic heat than a resting muscle (5 vs. 0.5 mW g^−1^). A high myocardial metabolic rate reflects a larger mitochondrial content in the cardiomyocytes (30–40% in small, and 15–20% in large animals) compared to skeletal muscle. It is estimated that the heart uses about 50% of its high energy consumption on basal metabolism and cross‐bridge activation, and only 15–20% consumption for contraction, that is, external work. This implies that about 80% of the myocardial oxygen consumption is spent on production of heat (Barclay et al., 2003; Baxi et al., 2000). It has been proposed that this seemingly ‘wasteful’ high basal heat production by the heart can be diverted to support the need for higher workloads, such as during aerobic exercise (Gibbs, 2003). The mechanical efficiency of skeletal muscle during exercise approximates that of the heart (∼35% when oxidative phosphorylation is the dominant metabolic pathway for ATP resynthesis; for example, Bangsbo et al., 2001; González‐Alonso et al., 2000). However, unlike the skeletal muscle, which is ‘built’ for strength of contraction, the myocardium develops only a fraction of the force, that is, 2−5 vs. 0.2 kg cm^−2^ cross‐section of muscle strip, respectively (Mommaerts, 1982). Detailed studies of the myocardial architecture have shown that cardiomyocytes are embedded in a dense meshwork of supporting collagen and elastic fibres. Enclosed by a fibrous pericardium, the myocardium is thus uniquely resistant to stretch (Anderson et al., 2005) and is protected against excessive filling and/or overdistension (Anderson et al., 2005) to which it is subject during various high output states. This is particularly important in the case of the thin‐walled right ventricle, but the condition can also affect the far less compliant left ventricle, for example, in takotsubo cardiomyopathy. Taken together, the above phenomena indicate that the heart plays a mediating role between the pulmonary and systemic circulations. Rather than being the power source for blood's propulsion, it assumes an autonomous, integrative function between the oxygen supply in the lung and its consumption at the periphery. Nonetheless, the heart's rhythmical filling and ejection clearly indicates a pump‐like action. The question arises what kind of a mechanical pump best demonstrates its operating principle.

## 6 HYDRAULIC RAM

**FIGURE 3:**
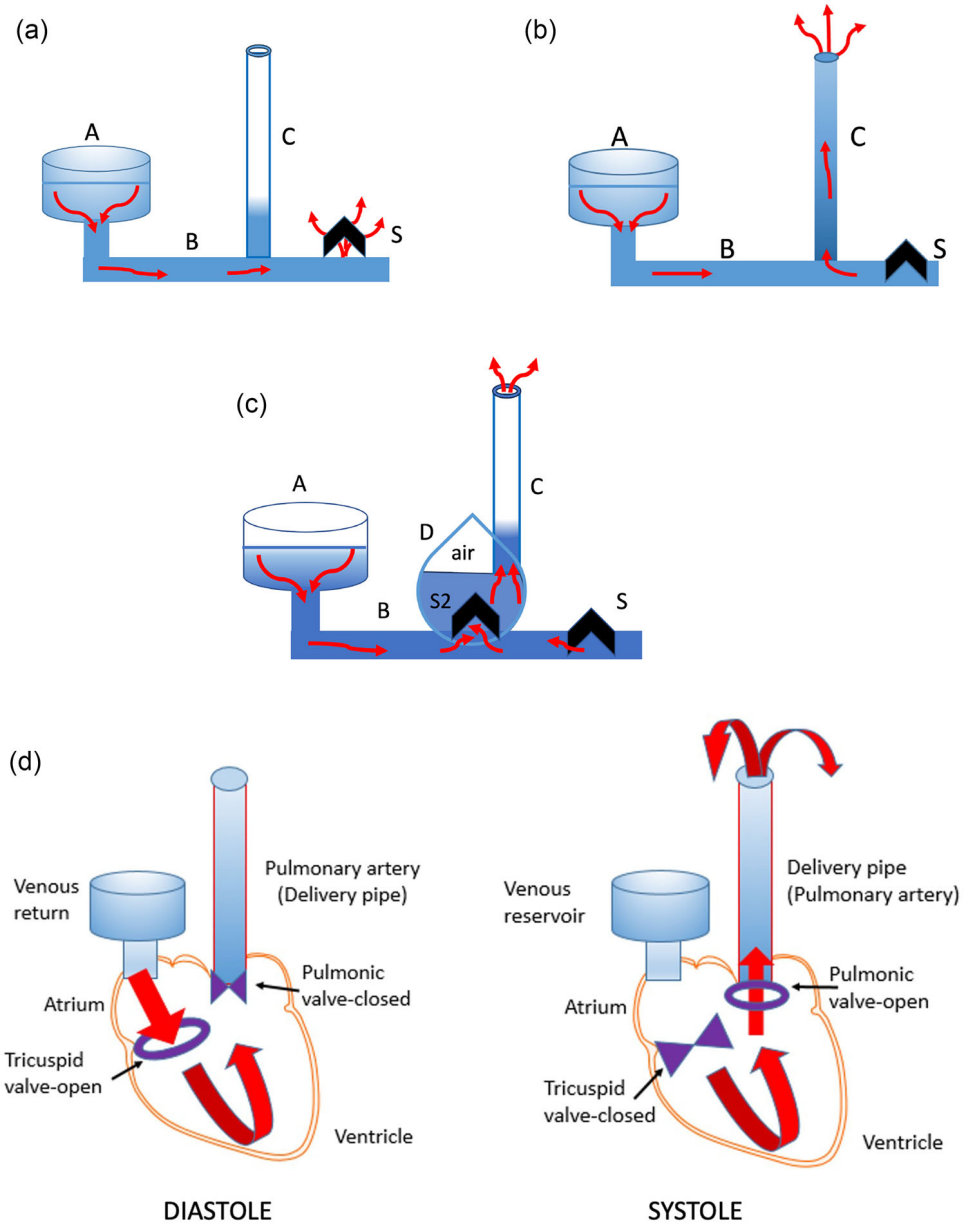
Components and working cycle of a hydraulic ram (a, b). (a) Water from the reservoir (A) accelerates by gravity along the drive pipe (B) and escapes from the open spill valve (S). (b) Drag from the accelerating water closes the spill valve (S), creating a back surge (water‐hammer effect) and an increase in pressure, forcing water to flow up the delivery pipe (C). A drop in pressure in the drive pipe (B) opens the spill valve (S) and the cycle repeats. (c) Automatic operation of a ram requires an additional valve (S2) and a pressure vessel (‘Windkessel’) (D). A build‐up of pressure (air cushion) in the pressure vessel (D) forces water to exit the delivery pipe (C). (d) Schematic representation of the heart as a hydraulic ram (right heart cycle). During diastole, blood flows from the atrium (reservoir) and fills the ventricle (analogous with (B) in the upper panel). In systole, flow reversal and build‐up of pressure in the ventricle close the tricuspid valve (analogous with spill valve (S)) and eject the blood into the pulmonary artery (delivery pipe (C)). (Adapted from Furst (2020a), used by permission of Springer‐Nature.)

In 1920, Steiner proposed that the heart has a ‘damming‐up’ function and that the principle of its action can be simulated by an impedance‐type water pump, a hydraulic ram (Steiner, 1999). The idea was further pursued by Havlicek (1937) and Manteuffel‐Szoege (1960), and more recently by Sengupta and Narula (2013) and Furst (2015, 2020a). As is in a hydraulic ram, the initial impulse for blood's movement in the isolated heart preparation is given by gravity (see Figure 3c). By ejecting the blood that is already in motion, the isolated heart preparation can thus be said to work according to the same conceptual principle as the ram. This concurs with the original formulation of Starling's law, namely, that the heart ‘pumps out all of the blood which it receives’ (Patterson & Starling, 1914). By assuming that the heart is the source of blood propulsion, the early researchers failed to recognize the similarities between the two operating principles (cf., Fig. 8 in Furst, 2015). If gravitational pressure energy is the cause of blood's motion in an isolated heart preparation, what forces might be responsible for the blood's movement in an intact organism?

## 7 THE SYPHON EFFECT

In clinical haemodynamics, the analogy with an electric circuit is commonly used whereby volume flow or CO is represented by the current, the pressure gradient between mean arterial (MAP) and central venous pressure (CVP) by voltage, and the peripheral resistance by a resistor. The systemic vascular resistance (*R*_sys_) can thus be obtained by using Ohm's law:

(3) $$R_{\mathrm{sys}}=\mathrm{MAP}-\mathrm{CVP}/\mathrm{CO}$$

It should be noted, however, that the parameter relationship given by Ohm's law is merely relational, without implying the causality. Unlike in an electrical circuit where the power source is known, and where voltage, current and resistance can be independently verified, in an intact circulation only CO and the pressure gradient (MAP − CVP) can be experimentally confirmed, whereas resistance (*R*_sys_) is a numerically derived value. The difficulty arises when a causal relationship existing in an electrical circuit is transposed to a clinical or experimental setting. Thus, one cannot assume a priori that the energy for the circulating blood is solely generated by the heart, and neither is it possible to measure the actual *R*_sys_ for which the number, dimensions, as well as the in‐ and outflow pressures of the patent capillaries and microvascular beds would have to be known. The problem of applying resistance to biological systems has been aptly addressed by Fishman:

The idea of resistance is unambiguous when applied to rigid tubes perfused by homogenous fluid flowing in a laminar stream … complexities are introduced when these concepts are extended to the pulmonary (as well as to systemic) circulation: the vascular bed is a non‐linear, visco‐elastic, frequency‐dependent system, perfused by a complicated non‐Newtonian fluid; moreover, the flow is pulsatile, so that the inertial factors, reflected waves, pulse wave velocity, and interconversions of energy become relevant considerations … as a result of many active and passive influences which may affect the relationship between the pressure gradient and flow, the term ‘resistance’ is bereft of its original physical meaning: instead of representing a fixed attribute of blood vessel, it has assumed physiological meaning as a product of a set of circumstances. (Fishman, 1963)

**FIGURE 4:**
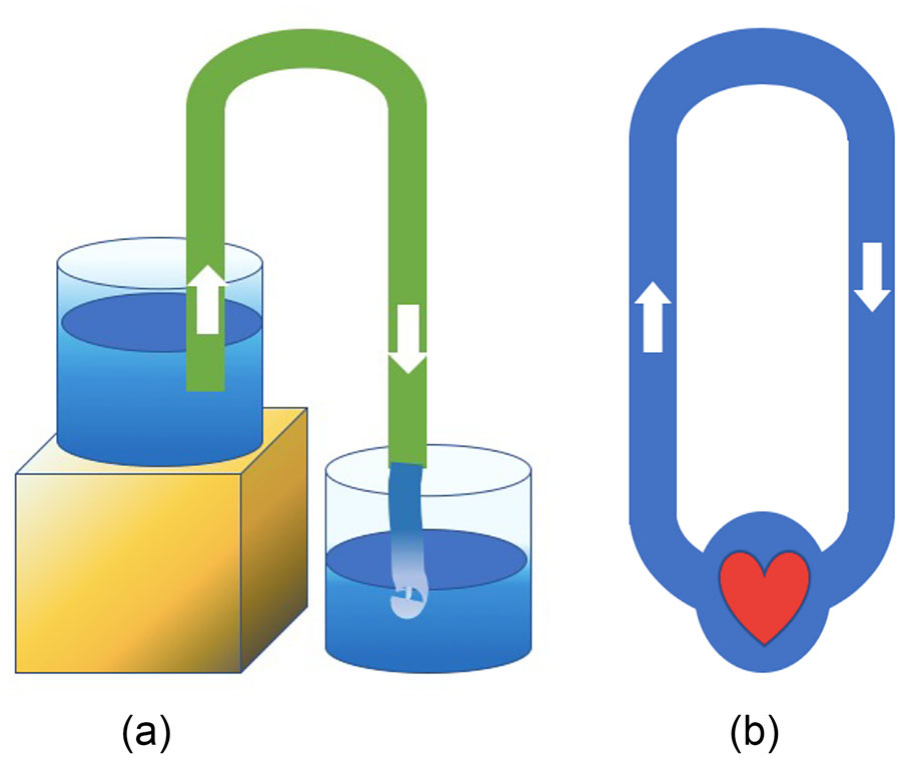
The siphon principle. (a) An open siphon is an inverted U‐shaped tube used to deliver a flow of liquid above the surface of the reservoir without a pump, powered by the combination of gravitational pressure and suction created within the tube. The principle of operation is based on Bernoulli's energy conservation law where, in an ideal liquid (at steady flow and without viscous losses) the sum of hydrostatic pressure, gravitational potential energy and kinetic energy remains constant. Once primed, the uphill flow of liquid in the shorter tube of the siphon is maintained by suction and is independent of the height of the loop. (b) Closed loop siphon. The circulatory system consists of numerous such loops *above* and *below* the level of the heart (inverted siphon). Counterbalance of forces in the ascending (arteries) and descending limbs (veins) eliminates the need for additional energy on the part of the heart to overcome gravity. (Reproduced from Furst (2020), used by permission of Springer‐Nature.)

The notion that the heart is the sole provider of energy for the circulating blood, moreover, disregards the basic laws of physics, namely, that a circular motion can only be maintained when the centrifugal and centripetal forces are in dynamic equilibrium. The former are conceptually represented by the arterial circulation, which distributes the blood to the periphery, and the latter by the venous system, which delivers the blood back to the centre, the right atrium. Considering that only about 15% of blood volume is in the high‐pressure, arterial compartment, 5% in the heart and 80% in a high‐compliance, low‐pressure venous compartment (Boulpaep, 2003a), such a system would be clearly unbalanced. Viewed from a dynamic perspective one would expect that the arterial limb of the circuit, tightly regulated by the baroreceptors and other neurohumoral control systems, would be closely matched by an equal and opposing field of negative tissue/intravascular pressure with its centre in the right atrium. Aukland and Reed have indeed proposed the existence of a differentiated peripheral regulatory system with ‘field‐like’ properties which would provide a local control of filtration/absorption at the level of the microcirculation in organs and tissues throughout the body (Aukland & Reed, 1993). This has been confirmed by human experiments in microgravity where the absence of gravitational hydrostatic pressure gradients causes the blood to shift into the chest and upper part of the body. Astronauts experience facial swelling and headaches due to increase in intracranial pressure (Hargens & Richardson, 2009). The application of a lower body negative pressure (LBNP) device counters some of these side effects. In view of the above, it can be implied that the blood circulates on the basis of the syphon principle (Figure 4). This operational principle has indeed been proposed but has not been adequately contextualized and is yet to gain a widespread acceptance (Hicks & Badeer, 1992).

## 8 THE SIGNIFICANCE OF NEGATIVE PERIPHERAL TISSUE PRESSURE

The interstitial space consists of an amorphous gel‐like ground substance, non‐organ specific cells, and collagen and elastic fibres organized in a reticuline mesh which holds water and small solutes. It makes up 15–25% of total body weight and the volume it occupies varies widely between 10% in skeletal muscle and 70% in loose connective tissue (Aukland & Nicolaysen, 1981). The majority of the interstitial space water is integrated into the ground substance and only a fraction is believed to be free. The hydrostatic pressure of this free fluid is defined as interstitial fluid pressure (IP) (Guyton et al., 1971). Radioisotope studies have demonstrated a dynamic exchange of water between the capillary membrane in the range of 80,000 litres per day (Boulpaep, 2003b). The discovery of membrane specific proteins in 1992, the aquaporins, involved in transmembrane diffusion of water and solutes, has shed new light on this highly dynamic and tightly controlled space. The aquaporins play a key role in the maintenance of cellular and tissue homeostasis during normal and pathological states. Experimental data have confirmed up to 50‐fold facilitation of aquaporin water transport across certain cell membranes (Day et al., 2014).

It was still assumed in the 1960s that IP was positive in reference to atmospheric pressure until Guyton and coworkers demonstrated, by way of an ingenious technique, that pressure in the interstitial space is in fact negative, in the range of −4 to −13 mmHg (Guyton et al., 1963). This appears to be the case in organs and tissues throughout the body, with the exception of the abdominal cavity and parenchyma of the liver. The average IP in a rabbit lung at functional residual capacity is −7.3 mmHg (Miserocchi et al., 1990). The IP is generated and maintained by a powerful draining action of the pulmonary lymphatics which under steady‐state conditions balances the net microvascular fluid filtration (Negrini et al., 1992). The lung is a highly perfused organ with the total number of lung capillaries in the order of 2.7 × 10^11^ (Weibel & Gomez, 1962). Considering the size and double blood supply of the lung, that is, functional and nutritional, the suctional forces generated by the lung parenchyma play an important role in maintaining the negative intrapleural pressure, which in turn influences venous return. Of note, the CVP in a sitting/upright human is invariably in the negative range (Dawson et al., 2004). We propose that the negative pressure generated by the lung parenchyma may provide additional forces which assist the flow of blood through the pulmonary vascular beds.

On the systemic side of the vascular circuit, the negative IP is essential for the maintenance of the normal skin turgor, a clinical sign of optimal vascular filling and tissue hydration. While even a minor tissue trauma will disturb the above‐mentioned forces and result in local tissue fluid collection (oedema), animal experiments have shown that visible systemic oedema requires a doubling of the interstitial fluid volume (Wiig & Reed, 1981). The intensity of suctional tissue forces can be readily demonstrated in an anaesthetized rat thermal injury model where burns of 10% of body surface area (BSA) are known to generate a negative IP of −20 mmHg and reach up to −30 mmHg in burns of 40% of BSA (Reed & Rodt, 1991). A mean tissue pressure of −135 mmHg has been reported in thermal injury experiments (Lund et al., 1988). Finally, the cause of the above‐mentioned tissue oedema and headward fluid shifts in microgravity environments has been ascribed to a drop in IP due to loss of tissue weight and gravitational loading (Hargens & Richardson, 2009). In summary, originating in a vast array of organ and tissue capillaries surrounded by zones of negative IP, the resultant force for venous return can be viewed as an independent factor. Guyton and other proponents of the venous return model aimed to prove this phenomenologically, but they could not get beyond the deeply ingrained cardiocentric view.

## 9 WHAT MOVES THE BLOOD?

The objection can be raised that a catastrophic drop in the arterial pressure due to ventricular fibrillation or asystole provides indisputable evidence that the circulation of blood depends solely on the ‘pumping action’ of the heart. While on the surface this appears to be the case, animal experiments have demonstrated that the blood continues to move after cardiac arrest, albeit at a rate which is incompatible with the preservation of higher‐organ function unless the rhythm is restored by resuscitative measures (Manteuffel‐Szoege et al., 1966; Skulec et al., 2018; Thompson, 1948). Rather than confirming the heart's propulsive function, such experiments point to blood's inherent property of self‐movement *in an intact organism*. The fact that a failing heart cannot maintain an adequate arterial pressure on account of myocardial damage, dysrhythmia or a malfunctioning valve does not support an a priori assumption that pressure generated by the heart is the *cause* of blood's propulsion; it rather points to its ram‐like function. While it is possible to separate the heart from the circulation for a limited period of time, such as during cardiopulmonary bypass, viewed physiologically, the heart and circulation comprise an inseparable unity and one cannot function without the other. Physiologist Adolf Jarisch made the following remark on the confounding issue of pressure: ‘For the development of the doctrine of the circulation it was undoubtedly fatal that the measurement of blood flow was comparatively laborious, but that blood pressure could be determined so easily. That is why the sphygmomanometer gained such a fascinating influence, although most organs do not need pressure, but flow (Jarisch, 1928)’.

Considering the heart functions like a hydraulic ram and the blood moves on the principle of a syphon, the only external work which the heart performs would be to eject the blood into the high pressure (arterial) compartment. As mentioned, an autonomous flow can readily be sustained by the syphon principle once the system has been primed. In a biological system the initial movement of the blood and the heart is ‘primed’ by the metabolic processes in the course of embryonic development and maintained throughout life. Non‐pressure‐driven ‘active’ fluid systems are well described in physics and may provide the mechanistic explanation for fluid transport in biological systems (Morozov, 2017; Wu et al., 2017). It has been demonstrated that self‐driven flows occur in a wide range of plant‐ and animal‐ microscopic conduits. The combination of electrostatic gradients between the negatively charged RBCs and the endothelial glycocalyx and the material exchange of solutes, water and heat across the capillary wall constitutes an essential requirement of a self‐driven fluid system. The presence of ubiquitous radiant energy, that is, external (exogenous) and internal (endogenous) heat loads, for instance, substantially increases flow velocity and provides additional driving force for the circulating blood (Koch Esteves et al., 2021; Li & Pollack, 2023). Recently, Li and Pollack repeated Maneuffel‐Szoege's classic chick embryo experiments (Manteuffel‐Szoege, 1960) and observed flow in the vitelline vessel for 50 min after arrest of the heart's action with KCl, though at a significantly slower rate; however, the post‐arrest flow velocity increased 3.7‐fold after exposing the preparation to infrared radiation (Li & Pollack, 2023). On the other hand, Koch Esteves et al. (2021) provided substantial evidence in the human leg that tissue blood flow during regional hyperthermia (local upper leg vs. lower leg heating) is controlled by highly localized events in the microcirculation, as opposed to central haemodynamic forces or thermal reflexes responding to increases in core temperature. Such phenomena point to the presence of an independent source of blood's movement at the level of the microcirculation which is intricately bound to local temperature and tissue metabolism.

Recent advances in microvascular research support that the RBCs – which account for 85% of all body cells (Sender et al., 2016) – function as oxygen and temperature sensors and modulators of microvascular tone (Ellsworth et al., 1995; Kalsi & González‐Alonso, 2012; Kalsi et al., 2017). The entrance of the RBCs into a region with increased metabolic demands, low pH, elevated temperature and increased sheer stress results in a drop in haemoglobin oxygen saturation and the release of nanomolar amounts ATP. The latter binds to endothelial purinergic receptors, causing the release of potent vasodilators, for example, nitric oxide and prostaglandins (e.g., Hearon et al., 2017; Mortensen et al., 2009). Significantly, the ensuing vasodilatation of the microvascular beds is conducted in the upstream direction (conducted vasodilatation) and acts as a positive feedback for local metabolic and thermoregulatory demands (Ellsworth *&* Sprague, 2012; González‐Alonso, 2012).

## 10 BLOOD CIRCULATION DURING EXERCISE

**FIGURE 5:**
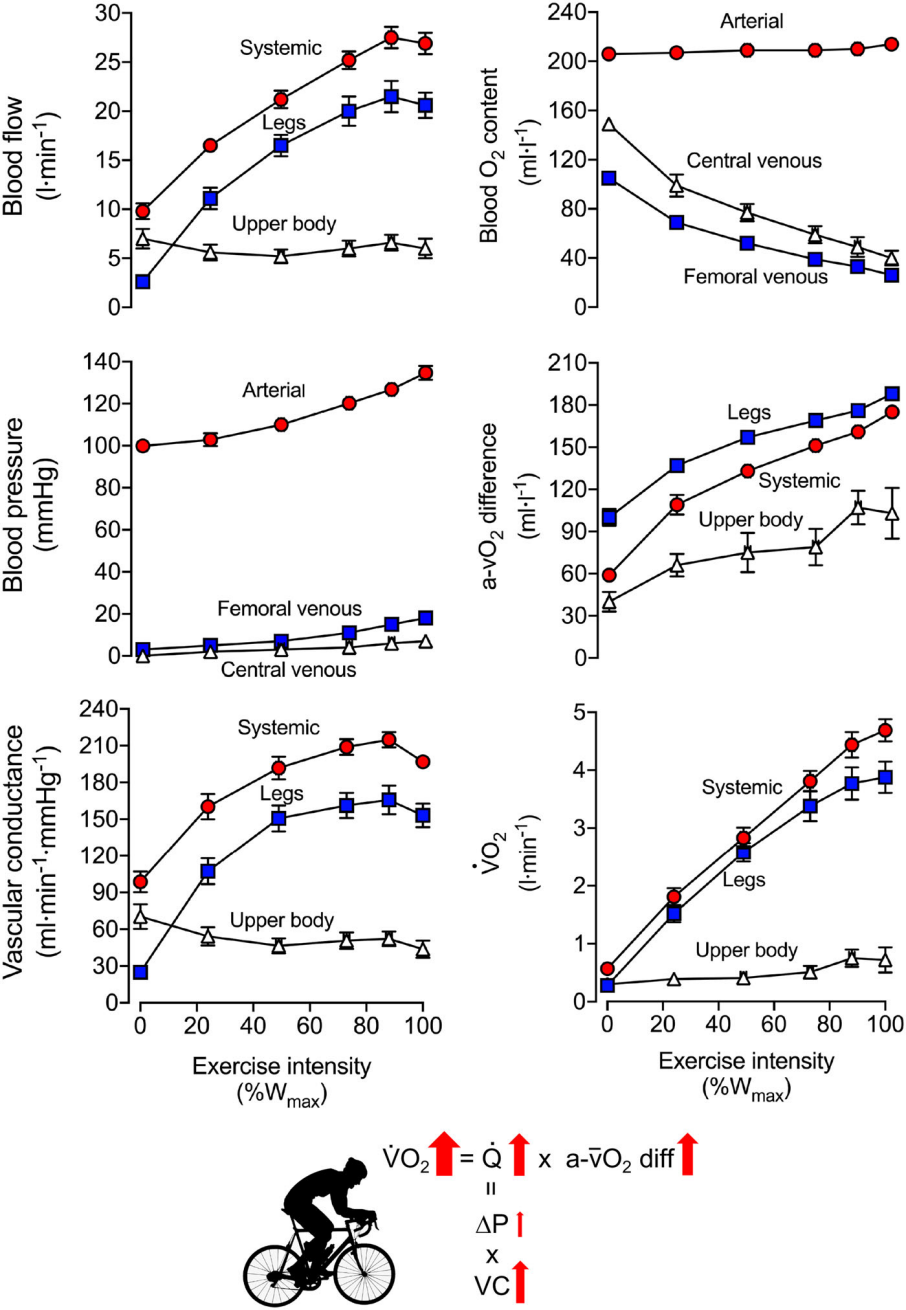
Regional blood flow, blood pressure, vascular conductance (VC), blood oxygen content, a‐vO_2_ differences and oxygen uptake (${\dot{V}}_{O_{2}}$) during incremental cycling exercise to exhaustion plotted against the relative exercise intensity (% of maximal power output; 460 ± 16 W). A 15 min submaximal exercise (120 W) and 3 min of seated rest preceded the incremental exercise protocol. Data are means ± SEM for 8–12 endurance‐trained male athletes with a mean ${\dot{V}}_{O_{2}\max}$ (±SD) of 4.7 ± 0.2 L min^−1^ or 57 ± 10 mL kg^−1^ min^−1^. Adapted from Mortensen et al. (2005 and 2008). Of note is the initial tight coupling between the increases in dynamically contracting muscle and systemic blood flow, and the dissociation between the several‐fold increase in systemic blood flow ($\dot{Q}$) and reductions or small changes in blood flow in the upper body tissues and organs.

**FIGURE 6:**
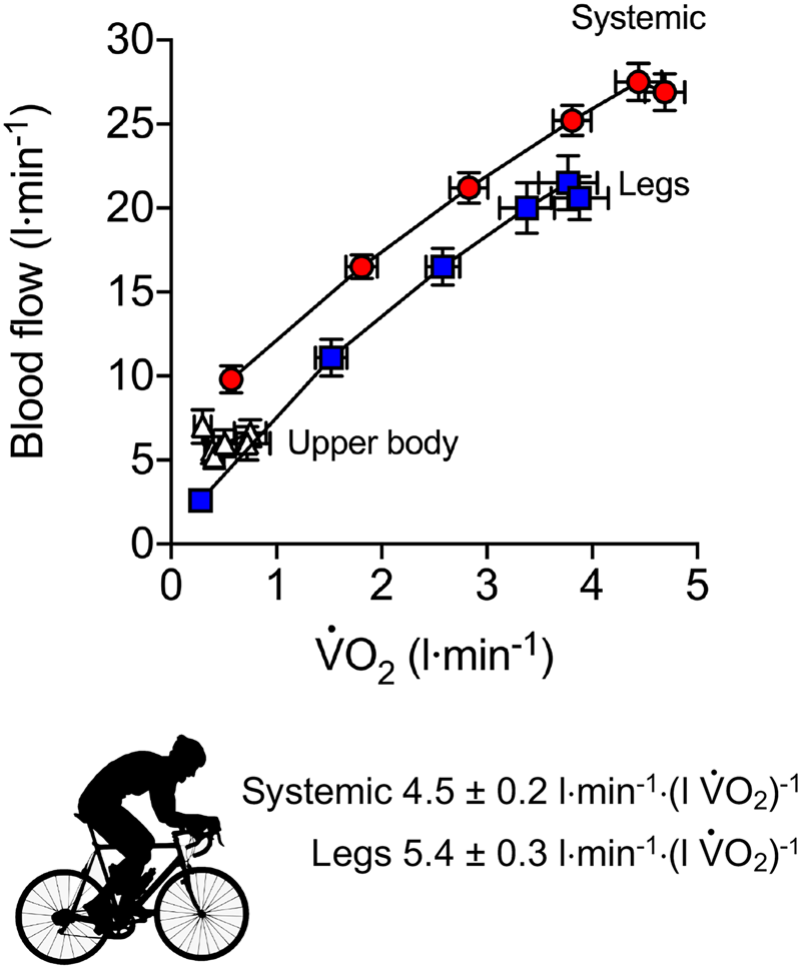
Blood flow and oxygen uptake relationships during incremental two‐legged cycling to exhaustion. Data were reported in Figure 5. Adapted from Mortensen et al. (2008). Significant linear relationships were observed between 0% and 90% of *W*_max_ with the mean slope being 4.5 ± 0.5 litres min^−1^ (l ${\dot{V}}_{O_{2}}$)^−1^ for the systemic blood flow and ${\dot{V}}_{O_{2}}$ and 5.4 ± 0.3 litres min^−1^ (l ${\dot{V}}_{O_{2}}$)^−1^ for the corresponding exercising legs comparisons. The increases in locomotor limb blood flow are largely associated with the elevations in local limb vascular conductance, indicative of changes in vessel diameter and blood viscosity (i.e., blood rheological properties).

Exercise‐induced stress can evoke the greatest stimulus for increasing peripheral and systemic blood flow (Joyner & Casey, 2015). Indeed, the integrative responses to exercise provide a key insight into the role of the heart and the periphery in the control of blood circulation (Joyner & Casey, 2015; Laughlin et al., 2012; Rowell, 1993; Travers et al., 2022). During incremental cycling in thermoneutral environments, locomotor muscle blood flow and CO increase according to elevated oxygen utilization (e.g., Calbet et al., 2007; González‐Alonso et al., 2015; Mortensen et al., 2008, 2005; Munch et al., 2014). In contrast, the blood flow in non‐exercising limbs and internal organs remains unchanged or declines (e.g., Calbet et al., 2007; González‐Alonso et al., 2015; Mortensen et al., 2008, 2005; Munch et al., 2014; Rowell et al., 1965, 1971; Vogiatzis et al., 2009). Because perfusion pressure changes relatively little, the increases in locomotor limb blood flow during graded exercise are proportional to the rise in regional vascular conductance (defined as peripheral blood flow per unit of pressure, the inverse of regional vascular resistance; Figure 5). Based on Poiseuille's law, the augmented vascular conductance is indicative of increased vessel dilatation and reductions in blood viscosity, factors known to be modulated by metabolic signals. In this context, the close coupling between blood flow and aerobic metabolism, amounting to 5–6 litres min^−1^ (l ${\dot{V}}_{O_{2}}$)^−1^ at the exercising legs and systemic circulation levels (Figure 6), points to a primary role of metabolically mediated locomotor muscle hyperaemia in the control of CO during exercise.

The dominant view, however, is that the heart is the primary regulator of CO and blood flow distribution by providing the pulsatile driving pressure for blood movement (Brengelmann, 2019; Levy, 1979; Reddi & Carpenter, 2005; Rowell, 1993; Secomb, 2016; Tyberg, 2002). In this model, cardiovascular function is predominantly determined by intrinsic factors, such as myocardial contractility, as well as extrinsic factors that alter the preload and afterload of the heart (i.e., venous return, pressure gradients and peripheral vascular conductance). Venous flow (venous return to the pump) is also driven by a pressure gradient between the venous vasculature of peripheral tissues and the right atrium and is facilitated by the action of the skeletal and respiratory muscle pumps (Brengelmann, 2019; Guyton, 1963; Miller et al., 2005; Rowell, 1993). When considering the dimensions of the human circulatory system, however, it is evident that the heart is small in comparison to the dimensions of the blood and blood vessels. The circulatory system of an adult human has on average a 245–331 g heart (3.8–4.3 g kg^−1^ body mass; Molina & DiMaio, 2012, 2015), 4.5–5.5 litres of blood (70–80 ml kg^−1^ body mass) (Oberholder et al., 2023) and 10,000–50,000 km of blood vessels (140–700 km kg^−1^ body mass) (Poole et al., 2020; Secomb & Pries, 2016). Should the human heart work as a conventional pressure‐propulsion pump, it would need to be much bigger to overcome the resistance to flow imposed by the 10,000–50,000 km total length of blood vessels, as most of them are microscopic capillaries which can be smaller than the RBCs (Ellsworth et al., 1995; Poole et al., 2020; Secomb & Pries, 2016). This is particularly relevant during exercise when the muscle contraction–relaxation cycle affects muscle blood flow dynamics (e.g., Miller et al., 2005; Rådegran, 1997).

The idea that the blood is primarily moved by contraction of the heart during exercise is also at odds with the observations that regional perfusion pressure is similar, but blood flow responses are different in the non‐exercising limbs and internal organs compared to the locomotor limbs (Calbet et al., 2004, 2007; González‐Alonso et al., 2015; Mortensen et al., 2008, 2005; Rowell et al., 1965, 1971). Moreover, the ‘cardiocentric’ model cannot satisfactorily explain why muscle and systemic blood flow does not increase further during maximal small muscle mass exercise (single leg knee‐extensor exercise or handgrip exercise) when the demand for blood flow is clearly below the cardiac functional capacity or why, during sprinting exercise, active muscle perfusion and cardiac output are below the cardiovascular capacity observed during maximal aerobic exercise (e.g., Calbet et al., 2015; Mortensen et al., 2008; Munch et al., 2014). The observation that increasing the heart rate by some 20 beats min^−1^ with right atrial pacing does not alter the circulatory responses to incremental aerobic exercise to exhaustion supports the view that factors associated with restrictions in peripheral (primarily active muscle) blood flow and vascular conductance – rather the alterations in myocardial function per se – are major determinants of the magnitude of total blood circulation (Munch et al., 2014). The metabolic, thermal, myogenic, mechanical and neurohumoral signals regulating active muscle perfusion and vascular conductance and thus the flow to the heart need to be considered to elucidate how the peripheral and central circulations are coupled across the full range of exercise intensities and modalities that humans can undertake, including exercise conditions evoking significant cardiovascular strain (e.g., González‐Alonso & Calbet, 2003; Joyner & Casey, 2015; Laughlin et al., 2012; Travers et al., 2022) or altering exercising limb blood flow and CO to precisely maintain O_2_ delivery to exercising muscles in response to profound alterations in blood oxygen content (e.g., Knight et al., 1992; González‐Alonso et al., 2006, 2002; Roach et al., 1999).

**FIGURE 7:**
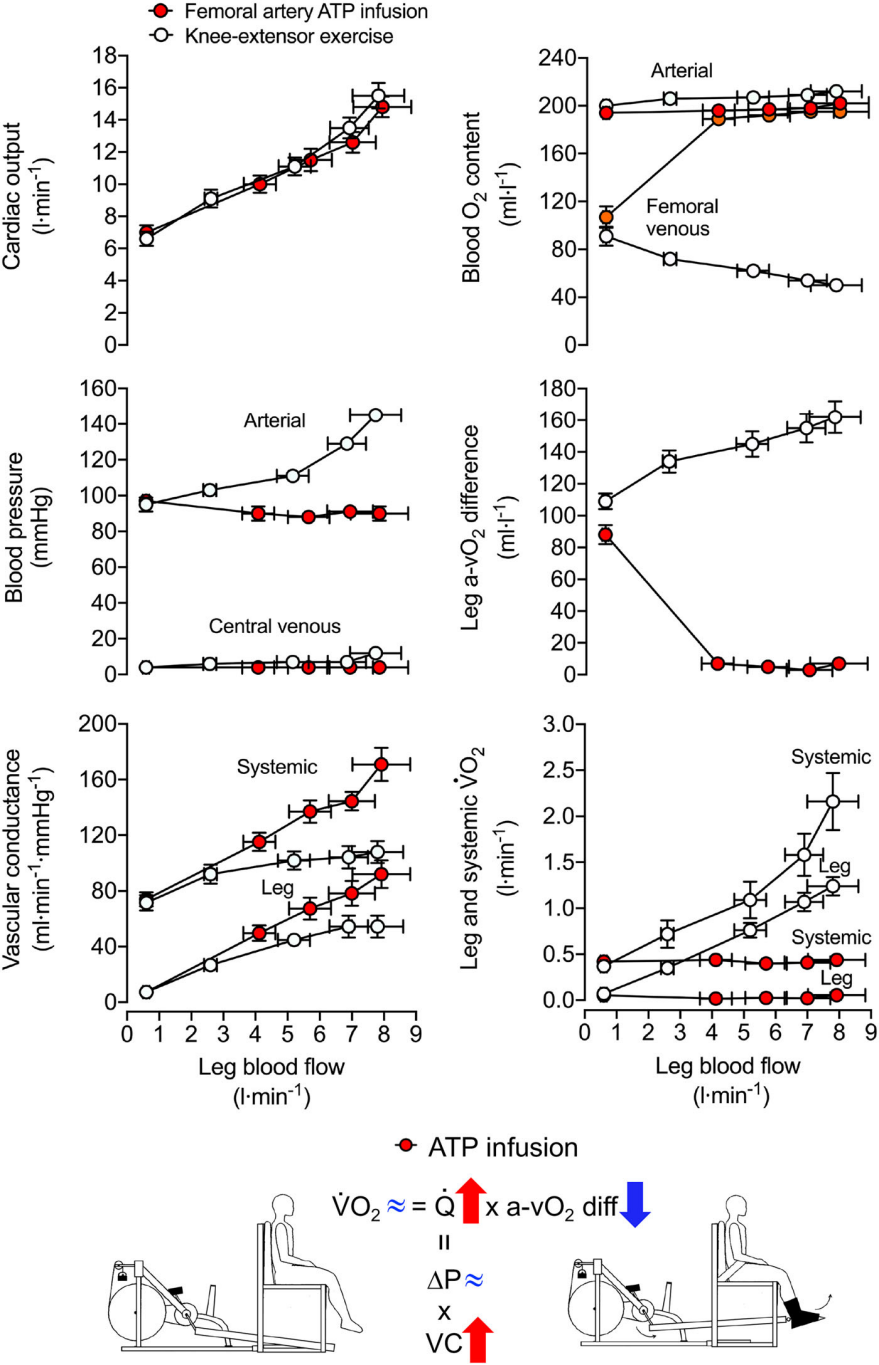
Leg and systemic haemodynamics with incremental one‐legged knee‐extensor exercise and graded intrafemoral artery ATP infusion. Cardiovascular variables are plotted against leg blood flow during incremental exercise and ATP infusion. Open cycles depict systemic haemodynamic responses and red circles depict leg haemodynamics. Data are means ± SEM for nine participants. Adapted from González‐Alonso et al. (2008). Intrafemoral artery ATP infusion induces the same increase in leg blood flow and cardiac output as single legged knee‐extensor exercise, but without increasing aerobic metabolism or (arterial and central venous) blood pressure. The increase in cardiac output ($\dot{Q}$) is therefore tightly linked to ATP‐induced increases in arterial and venous leg blood flow. ∆*P*, arterio–venous pressure gradient; VC, vascular conductance.

An alternative view to the ‘cardiocentric’ model is that events in the peripheral circulation play an important role in the control of CO and that the activity of the heart per se has a permissive effect on CO (Furst, 2020a; Krogh, 1912a, 1912b; Travers et al., 2022; Watanabe et al., 2020). In this context, human investigations using pharmacologically induced limb vasodilatation (via intra‐arterial infusion of ATP and other nucleotides) have shown profound increases in CO in proportion to the increase in limb blood flow (up to ∼7–8 litres min^−1^ increase in leg blood low and CO from baseline without meaningful alterations in central venous or arterial pressure: Figure 6; González‐Alonso et al., 2008; Rosenmeier et al., 2008), whereas limb vasoconstriction (via combined intra‐arterial infusion of adenosine and the sympathomimetic agent tyramine or the combined blockade of prostaglandins and nitric oxide using *N* ^G^‐monomethyl‐l‐arginine and indomethacin infusion) leads to a proportionate decrease in limb blood flow and CO (Mortensen et al., 2007; Rosenmeier et al., 2008). Moreover, the combination of elevations in heart rate with right atrial pacing and limb tissue vasodilatation with either femoral artery ATP infusion or single leg knee‐extensor exercise does not alter systemic or peripheral (leg and brain) blood flow (Bada et al., 2012). This raises the question whether vasodilator and vasoconstrictor substances modulate limb blood flow and CO by acting on specific receptors located in the arterial system or the venous vasculature and the heart. The finding that comparable infusion of ATP in the femoral vein does not change limb blood flow or CO in resting humans (González‐Alonso et al., 2008) suggests that the ATP‐mediated signalling transduction mechanisms, which so markedly increase limb blood flow and CO when ATP is infused in the femoral artery, originate in the microcirculation, not in the large veins or the heart itself. As pharmacologically induced vasodilatation in the resting leg does not alter metabolism or arteriovenous blood pressure gradients, the study also revealed that activation of the skeletal muscle pump, changes in muscle metabolism or alterations in perfusion pressure are not obligatory for sustaining venous return, CVP, stroke volume and CO or maintaining muscle blood flow during exercise in humans (Figure 7). These observations collectively lend support to the idea that local skeletal muscle mechanisms inducing increases in locomotor limb tissue blood velocity and flow largely determine venous flow to the heart and thus the coupling of the peripheral and central circulations during exercise. Whether the increases in arterial and venous blood velocity and flow in exercising muscle are mediated via changes in blood vessel diameter (vasodilatation), alterations in the blood's rheological properties (e.g., decreases in blood viscosity and increases in RBC deformability and dispersion) and/or other yet‐to‐be‐identified mechanisms warrants future investigation.

## 11 CONCLUSIONS

This review highlights a long‐standing debate between the proponents of the ‘cardiocentric’ view, who maintain that the heart is the main controller of CO, and the adherents of the ‘venous return’ view, where the heart plays a secondary role to the peripheral circulation. It is argued that the debate cannot be resolved because both views assume that the pressure‐generating action of the heart is the sole source of blood's movement. Examples from the field of experimental physiology and clinical medicine show that pressure‐propulsion models are unable to account for an increasing number of circulatory phenomena and clinical treatment modalities. For example, the developmental biology of the cardiovascular system shows that the blood circulates prior to functional maturity of the heart, and its movement is intricately connected with the metabolic demands of the tissues. Thus, the flow is primary and the pressure maintained by the heart is a later gain of vertebrate evolution. An alternative, ‘integrative’ circulation model is proposed by which the forces for the circulating blood result from the dynamic equilibrium between the centrifugally acting arterial pressure sustained by the heart, and the centripetally acting, suctional forces generated by the metabolic activity of organs and tissues and related factors such as local temperature and blood oxygen levels. Rather than working as a hydraulic pump, the heart in this model functions on the principle of a hydraulic ram, ejecting ‘all of the blood it receives’ (Patterson & Starling, 1914). Finally, the circulatory responses to exercise and pharmacologically induced blood flow alterations are discussed to support of the need for an alternative circulation model to the conventional ‘cardiocentric’ view.

## AUTHOR CONTRIBUTIONS

Branko Furst proposed the original draft of the review and designed the first manuscript. Branko Furst and José González‐Alonso wrote the subsequent drafts and final manuscript. Branko Furst made Figures 1, 2, 3, 4 and José González‐Alonso Figures 5, 6, 7. Both authors made critical revisions and approved the final version of the manuscript. Both authors agree to be accountable for all aspects of the work in ensuring that questions related to the accuracy or integrity of any part of the work are appropriately investigated and resolved. All persons designated as authors qualify for authorship, and all those who qualify for authorship are listed.

## CONFLICT OF INTEREST

The authors declare no conflicts of interest.

## FUNDING INFORMATION

No funding was received for this work.
